# Transient Diabetes Insipidus With Pituitary Dysfunction Following Abdominal Surgery: A Rare Postoperative Endocrine Complication

**DOI:** 10.7759/cureus.97148

**Published:** 2025-11-18

**Authors:** Parvathy Subhash, Ajith Kurien, Michelle Lim

**Affiliations:** 1 Internal Medicine, Peterborough City Hospital, Peterborough, GBR; 2 Nephrology, Peterborough City Hospital, Peterborough, GBR

**Keywords:** abdominal surgery, diabetes insipidus, hypogonadotropic hypogonadism, pituitary dysfunction, postoperative complications

## Abstract

Diabetes insipidus (DI) developing postoperatively is a recognized complication following neurosurgical procedures involving the hypothalamic-pituitary region. However, its occurrence after non-neurosurgical operations is exceedingly rare. We report a case of polyuria associated with hypogonadotropic hypogonadism, closely resembling central DI, following major abdominal surgery. Diagnosing DI after non-neurosurgical procedures is challenging, as polyuria is often attributed to more common causes, such as osmotic diuresis or fluid overload. Awareness of this rare but significant postoperative complication is essential for prompt diagnosis and appropriate multidisciplinary management.

## Introduction

Diabetes insipidus (DI) is a rare but treatable disorder, with a prevalence of approximately 1 in 25,000 individuals [1]. Central DI results from insufficient secretion of arginine vasopressin (AVP) from the posterior pituitary, whereas nephrogenic DI is characterized by resistance to ADH action at the kidneys. Central DI is more common and is typically associated with hypothalamic or pituitary tumors, neurosurgical procedures, trauma, or infiltrative diseases [2]. Nephrogenic DI has been linked to drugs such as lithium and conditions like hypercalcemia [3,4]. The development of DI following non-neurosurgical procedures is exceedingly rare. In such cases, postoperative polyuria is often attributed to more common causes, including osmotic diuresis, fluid overload, or psychogenic polydipsia [5]. Massive blood loss has been proposed as a potential cause of posterior pituitary ischemia, leading to impaired ADH synthesis and release [6]. We present a rare case of transient DI with concurrent hypogonadotropic hypogonadism following major abdominal surgery, highlighting the importance of recognizing uncommon endocrine complications in the postoperative period.

## Case presentation

A 47-year-old male with type 2 diabetes mellitus, peripheral vascular disease, and obesity underwent an open abdominoperineal resection with end colostomy for rectal carcinoma. He had no prior history of surgical procedures. His regular medications included apixaban, atorvastatin, metformin, omeprazole, and aspirin. General anesthesia was induced without complication; however, the patient experienced approximately one hour of intraoperative hypotension, requiring metaraminol infusion.

Postoperatively, he was admitted to the intensive therapy unit for observation and was later stepped down to the surgical ward. Within 48 hours, he developed pronounced polyuria and polydipsia. Urine output exceeded 3,000 mL/day, peaking at 6,800 mL/day, with oral fluid intake up to 5,700 mL/day. No similar symptoms had been reported preoperatively. Laboratory results showed a mild rise in creatinine (from a baseline of 90 µmol/L to 120 µmol/L), which was disproportionate to the degree of polyuria.

**Figure 1:**
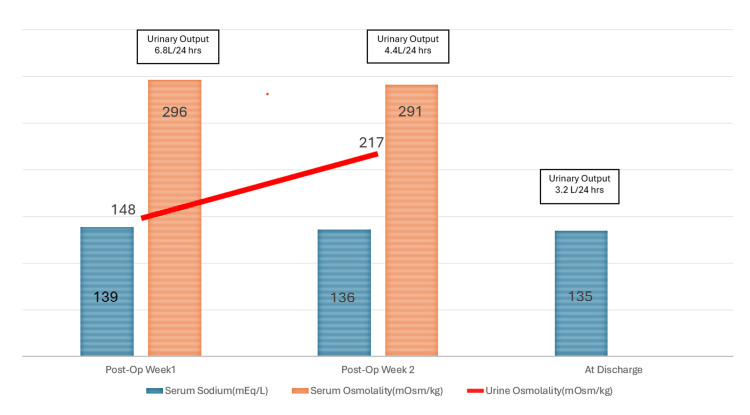
Trends of serum sodium and osmolality in relation to urine output throughout admission

**Figure 2:**
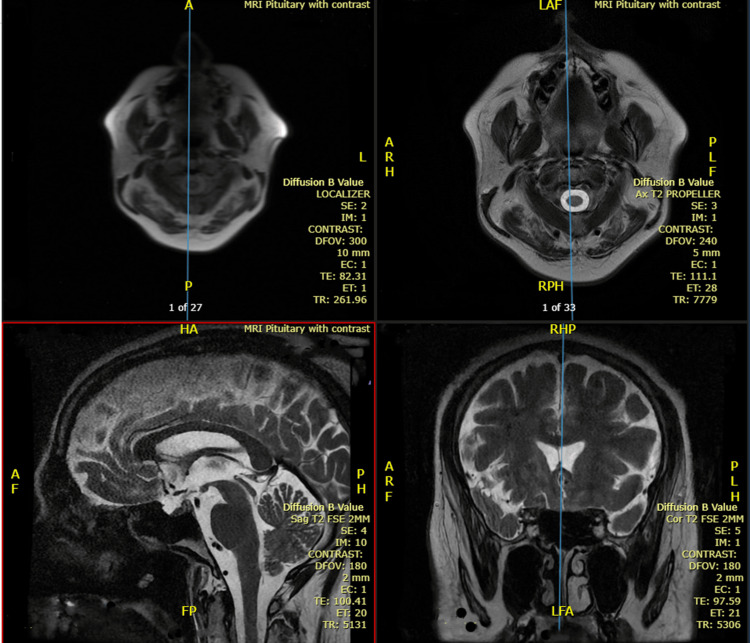
MRI of the brain in sagittal and coronal views showing normal brain parenchyma

Blood glucose levels remained stable, effectively excluding osmotic diuresis. Serum sodium was normal (139 mmol/L), and preliminary investigations revealed normal electrolytes, including calcium (Table 1). Despite cautious fluid restriction, polyuria persisted. An endocrinology consultation was obtained. Investigations demonstrated elevated serum osmolality (296 mOsm/kg) with inappropriately dilute urine (148 mOsm/kg), consistent with DI (Figure 1). Hormonal assessment revealed low testosterone (4.5 nmol/L) and low sex hormone-binding globulin (11.9 nmol/L) with normal luteinizing hormone and follicle-stimulating hormone, indicative of hypogonadotropic hypogonadism. Correlating the clinical symptoms with the abnormal hormone panel suggested that central DI was the most likely etiology. MRI of the pituitary gland was normal (Figure 2).

**Table 1:** Baseline blood test results

Test	Result
Sodium	139 mmol/L
Urine sodium	40 mmol/L
Creatinine	112 µmol/L (baseline: ~90)
Potassium (serum)	3.8 mmol/L
Calcium (serum)	2.15 mmol/L

With conservative management, including gradual fluid restriction to 3 L/day and close biochemical monitoring, the patient’s urine output began to normalize. Urine osmolality increased to 217 mOsm/kg, and serum osmolality improved to 291 mOsm/kg. Serum sodium remained within the normal range, and renal function returned to baseline. The patient was discharged with arrangements for outpatient endocrine follow-up.

## Discussion

Central DI following non-neurosurgical procedures is extremely rare. Most cases of postoperative DI are associated with direct pituitary or hypothalamic injury, whereas this case likely represents transient hypothalamic-pituitary dysfunction secondary to intraoperative hypotension or stress-related suppression of AVP release [7]. The concurrent transient hypogonadotropic hypogonadism suggests reversible hypothalamic-pituitary dysfunction rather than isolated posterior pituitary injury. Postoperative polyuria can easily be attributed to other mechanisms, including osmotic diuresis and fluid overload. Diagnostic evaluation should include paired serum and urine osmolality measurements, assessment of sodium and renal function, and exclusion of other metabolic causes. Early endocrine involvement is critical to confirm a suspicion of DI and guide treatment. In this case, spontaneous resolution occurred within days, supporting a transient and reversible hypothalamic-pituitary disturbance.

## Conclusions

Transient central DI is a rare but clinically significant postoperative complication, even in the absence of neurosurgical intervention. This case highlights the importance of maintaining vigilance for endocrine causes of postoperative polyuria, particularly when common metabolic and renal explanations have been excluded. Early recognition and timely multidisciplinary management are essential to prevent complications and optimize patient outcomes.
